# Supplementary material for: Elevational patterns of the percentages of plant genera with tropical and temperate affinities in Nepal
Source: PeerJ. 2019 Feb 8;7:e6116. doi: 10.7717/peerj.6116 (PMC6369827; doi:10.7717/peerj.6116)
Supplement: Supplemental Information 1 [file peerj-07-6116-s001.doc]

| Responses | Predictors | nVars | Adjusted R^2^ | AIC_c_ | ΔAIC_c_ |
| --- | --- | --- | --- | --- | --- |
| The percentages of tropical genera | MTCM, MAP, PSN^**^ | 3 | 0.998 | -66.9 | 1.7 |
|  | MTCM, MAP* | 2 | 0.988 | -68.6 | 0 |
|  | MAP, PSN | 2 | 0.918 | 34.8 | 103.4 |
|  | MTCM, PSN | 2 | 0.986 | -62.1 | 6.5 |
|  | MTCM | 1 | 0.986 | -62.6 | 6 |
|  | MAP | 1 | 0.913 | 36.6 | 105.2 |
|  | PSN | 1 | 0.714 | 101.1 | 169.7 |
| The percentages of temperate genera | MTWM, PWETQ, PSN, TEPMDE | 4 | 0.96 | -61.1 | 2.4 |
|  | MTWM, PWETQ, PSN | 3 | 0.934 | -35.8 | 27.7 |
|  | MTWM, PWETQ, TEPMDE | 3 | 0.96 | -60.8 | 2.7 |
|  | MTWM, PSN, TEPMDE | 3 | 0.962 | -63.5 | 0 |
|  | PWETQ, PSN, TEPMDE | 3 | 0.913 | -20.9 | 42.6 |
|  | PSN, TEPMDE | 2 | 0.916 | -23.3 | 40.2 |
|  | MTWM, TEPMDE | 2 | 0.955 | -57.3 | 6.2 |
|  | PWETQ, TEPMDE | 2 | 0.915 | -23.3 | 40.2 |
|  | MTWM, PWETQ | 2 | 0.889 | -8.0 | 55.5 |
|  | PWETQ, PSN | 2 | 0.881 | -5.3 | 58.2 |
|  | MTWM, PSN | 2 | 0.93 | -32.8 | 30.7 |
|  | MTWM | 1 | 0.877 | -6.0 | 57.5 |
|  | PWETQ | 1 | 0.846 | 7.4 | 70.9 |
|  | PSN | 1 | 0.863 | 1.065 | 64.565 |
|  | TEPMDE | 1 | 0.265 | 91.7 | 155.2 |

**Support Information**

S1 Adjusted coefficients of determination (R^2^) and the corrected Akaike information criterions (AIC_c_) of all possible multiple regressions models. There were 7 and 15 possible multiple regressions models for tropical and temperate percentages, respectively. *, predictors in the first best model; **, predictors in the second best model.
